# FACTORS ASSOCIATED WITH NURSING HOME DIRECT CARE PROFESSIONALS’ TURNOVER INTENT DURING COVID-19

**DOI:** 10.1093/geroni/igac059.1964

**Published:** 2022-12-20

**Authors:** Verena Cimarolli, Natasha Bryant, Francesca Falzarano, Robyn Stone

**Affiliations:** LeadingAge, Washington, District of Columbia, United States; LeadingAge, Washington, District of Columbia, United States; Weill Cornell Medicine, Douglaston, New York, United States; LeadingAge, Washington, District of Columbia, United States

## Abstract

The negative effects of the COVID-19 pandemic on the well-being of direct care professionals (DCPs; nursing assistants and aides) in nursing homes (NHs) has led to high rates of DCPs’ turnover and staff shortages - both issues that were already prevalent before the pandemic. More optimal staffing levels and less turnover are essential for optimal NH quality of care, but little is known about factors associated with turnover in DCPs in NHs during the pandemic. Hence, the purpose of this study was to examine the mediating roles of 1) quality of employer communication related to COVID-19; 2) DCPs’ perceived preparedness to care for residents with COVID-19; and 3) DCPs’ job satisfaction in the relationship between overall COVID-19-related work stress and intent to remain in one’s job (an indicator of turnover). Path analyses (N=809) demonstrate a significant, indirect effect between COVID-19-related stress and intent to remain in one’s position through the variables of communication, preparedness, and job satisfaction. Higher levels of COVID-19 related stress were associated with poorer communication quality, lower levels of preparedness, and lower job satisfaction, which was subsequently associated with a reduced likelihood of intent to remain in one’s job. However, direct effects show that better communication quality was associated with better preparedness correlating with higher job satisfaction which increased the likelihood of intent to remain in one’s job. Findings underscore the importance of employer supports in DCPs’ job satisfaction and turnover in NHs and, thus have implications for how to improve quality of care in NHs.

